# Markers of Oxidative Stress and Inflammation in Ascites and Plasma in Patients with Platinum-Sensitive, Platinum-Resistant, and Platinum-Refractory Epithelial Ovarian Cancer

**DOI:** 10.1155/2017/2873030

**Published:** 2017-08-07

**Authors:** Juan Carlos Cantón-Romero, Alejandra Guillermina Miranda-Díaz, Jose Luis Bañuelos-Ramírez, Sandra Carrillo-Ibarra, Sonia Sifuentes-Franco, José Alberto Castellanos-González, Adolfo Daniel Rodríguez-Carrizalez

**Affiliations:** ^1^Hospital of Gynecology and Obstetrics, Department of Oncology Gynecology, Sub-Specialty Medical Unit, National Occidental Medical Center, Mexican Social Security Institute, Guadalajara, JAL, Mexico; ^2^Institute of Experimental and Clinical Therapeutics, Department of Physiology, University Health Sciences Centre, University of Guadalajara, Guadalajara, JAL, Mexico; ^3^Specialties Hospital, National Occidental Medical Centre, Mexican Social Security Institute, Guadalajara, JAL, Mexico

## Abstract

Diverse proinflammatory biomarkers and oxidative stress are strongly associated with advanced epithelial ovarian cancer (EOC). *Objective*. To determine the behavior of markers of oxidative stress and inflammation in plasma and ascites fluid in patients with platinum-sensitive, platinum-resistant, and platinum-refractory EOC. *Methods*. A prospective cohort study. The colorimetric method was used to determine levels of the markers 8-isoprostanes (8-IP), lipid peroxidation products (LPO), and total antioxidant capacity (TAC) in plasma and ascites fluid; and with ELISA, the levels of interleukin-6 (IL-6) and tumor necrosis factor alpha (TNF-*α*) were determined in patients with EOC. *Results*. In ascites fluid, a significant increase in 8-IP versus baseline plasma levels was found (*p* = 0.002). There was an important leakage of the TAC levels in ascites fluid versus baseline plasma levels (*p* < 0.001). The IL-6 was elevated in ascites fluid versus baseline plasma levels (*p* = 0.003), and there were diminished levels of TNF-*α* in ascites fluid versus baseline plasma levels (*p* = 0.001). *Discussion*. We hypothesize that the ascites fluid influences the behavior and dissemination of the tumor. Deregulation between oxidants, antioxidants, and the proinflammatory cytokines was found to vary among platinum-sensitive, platinum-resistant, and platinum-refractory patients.

## 1. Introduction

The risk of developing epithelial ovarian cancer (EOC) in females > 65 years old fluctuates ~0.36% in developing countries and 0.64% in developed countries, which makes EOC very frequent in women [1]. In Europe, little more than one-third of women with EOC survive five years after diagnosis because the majority are diagnosed in advanced stages [2]. Globally, about 75% of cases are diagnosed at stages III and IV [3]. The hypothetical theory of incessant ovulation suggests that repeat ovulation is responsible for the epithelial transformation of the ovaries because the epithelial cells that surround the zone where the follicular rupture occurred are exposed to mutagenic mediators of inflammation during the preovulatory period, with the capacity to produce genomic damage conducive to apoptosis and the excessive production of inflammation and oxidative stress [4]. However, recent studies have shown that EOC does not always present the typical characteristics of the mesodermal epithelium, which brings forth the hypothesis that the EOC originates in the fallopian tubes in the form of inclusion cysts that may or may not be present in the cancerous state [5, 6].

The majority of women with EOC have a high grade of malignancy, and ~84% are found in stage IIIC. The EOC spreads across the peritoneal surface affecting the pelvic and abdominal cavity. Stage IV (12–21%) is characterized by distal metastasis (hepatic/splenic) and extra-abdominal metastasis [7]. Malignant ascites has gained recognition as a unique form of tumor environment responsible for the characteristics of EOC. Ascites is considered an important component for tumor progression [8]. The link between the presence of ascites and the progression of EOC was proposed by Rocconi et al., and since then, numerous studies have contributed to the categorization of the components of ascites, revealing the importance of its role in EOC [9]. The cellular components of ascites contain an ample and complex, heterogeneous mix of cell populations, including tumoral and stromal cells, each one with a defined role, including fibroblasts, endothelial or mesothelial cells, adipocytes, stromal cells derived from adipose tissue, stem cells derived from bone marrow, and immune cells [10]. Some of the cellular components of stroma cells are capable of activating the vascular endothelial growth factor (VEGF) [11]. Ascites is an inflammatory fluid that can be produced in large quantities in EOC. One recent study reported that the IL-6 is strongly associated with advanced EOC and that the IL-6 findings could be useful in combination with serum levels of CA-125 to differentiate between benign tumors and EOC [12]. A study published in 2013 reported significantly increased levels of the marker of oxidative DNA damage (8-hydroxy-2-deoxyguanosine) and the 8-isoprostanes (a marker of oxidative stress) in peritoneal fluid in women with severe endometriosis [13]. Thus, it is of interest to study the behavior of diverse proinflammatory biomarkers (IL-6 and TNF-*α*) and oxidative stress (products of lipid peroxidation, 8-isoprostanes, and the total antioxidant capacity) in plasma and in ascites fluid in patients with EOC.

In the standard treatment for locally advanced EOC in stages III and IV [14] with criteria of inoperability due to carcinomatosis, it is recommended to administer tricyclic neoadjuvant platinum-based chemotherapy and taxanes, followed by intervals of surgery and consolidation with platinum-based chemotherapy [15]. When cytoreduction is not feasible, neoadjuvant therapy is recommended in patients sensitive to the medications, and afterwards, they will undergo cytoreductive surgery [16]. The election of chemotherapy is actually based, in part, on the duration and type of response to initial therapy: for platinum-sensitive illness (an interval free of disease progression ≥ 6 months from the end of the taxane/platinum treatment) and for platinum-resistant illness (<6 months), nonplatinum regimens are used: liposomal pegylated doxorubicin, topotecan, gemcitabine, etoposide, and taxanes, which have been demonstrated to have similar efficacy and acceptable for use in these patients [17]. Another management alternative for platinum-resistant patients is the bevacizumab. The bevacizumab is a recombinant humanized monoclonal antibody with antiangiogenic effect that binds with all of the isoforms of the vascular endothelial growth factor (VEGF). It is approved by the European Medicines Agency as a treatment for the first recurrence of platinum-sensitive EOC and for the management of various solid tumors in combination with cytotoxic chemotherapy [18]. Women who present with progression despite the platinum are considered platinum-refractory and present with the worst prognosis [19].

The objective of the study was to determine the behavior of markers of oxidative stress and inflammation in plasma and ascites fluid in platinum-sensitive, platinum-resistant, and platinum-refractory EOC patients.

## 2. Materials and Methods

In a prospective cohort with 12 months of follow-up, all females who attended the Hospital of Gynecology and Obstetrics, Department of Oncology and Gynecology, at the National Occidental Medical Centre of the Mexican Social Security Institute in Guadalajara, Jalisco, Mexico, who had ascites fluid and a preoperative diagnosis of EOC, and who agreed to sign the informed consent form, were included. Not included were minors whose parents or guardians did not agree for them to participate in the study, those who had antecedents of cancer in another organ or system, those who had received chemotherapy previously, or adult patients who did not agree to sign the informed consent.

A 5 mL baseline blood sample and a 2 mL sample of ascites fluid were obtained before the onset of chemotherapy. After 12 months, another blood sample (5 mL) was obtained. We included the plasma of 6 healthy women who came for a regular visit with the gynecologist and the data served to establish the normal levels of the reagents.

### 2.1. Biochemical Analysis

The blood samples were collected with 0.1% of ethylenediaminetetraacetic (EDTA). The plasma and ascites fluid were separated by centrifugation at 2000 rpm for 10 min at room temperature and stored at −80°C until processing. All technical readings of optical density were made with the Synergy HT (BioTek®) microplate reader.

### 2.2. TNF-*α* and IL6

The IL-6 and TNF-*α* levels were determined by ELISA, following the instructions of the kit manufacturer (PeproTech®, Rocky Hill, NJ 08553, USA). Both cytokines had a detection limit of 32 pg/mL. First, 100 *μ*L of diluted capture antibody was added, followed by incubation overnight at room temperature. Then, 300 *μ*L of blocking buffer was added to the wells and it was incubated for 1 h at room temperature. Plasma or ascites fluid and standards were added, followed by incubation for 2 h at room temperature. After several washings, 100 *μ*L of diluted detection antibody was added and incubated at room temperature for 2 h. Then, 100 *μ*L diluted HRP-avidin conjugate was added, followed by incubation for 30 min at room temperature. Finally, 100 *μ*L of substrate solution was added to each well. The plate was read at a wavelength of 405 nm with correction set at 650 nm and was reported in pg/mL. The TNF-*α* intra-assay coefficient of variation (CV) was 2.1%, and the intra-assay CV for IL-6 was 4.7%.

### 2.3. Products of Lipid Peroxidation

The levels of lipoperoxides (LPO) in plasma and ascites fluid were measured using the FR22 assay kit (Oxford Biomedical Research Inc., Oxford, MI, USA) according to the manufacturer's instructions. The limit of detection for this test was 0.1 nmol/mL. In this assay, the chromogenic reagent reacts with malondialdehyde (MDA) and 4-hydroxy-alkenals to form a stable chromophore. First, 140 *μ*L of plasma or ascites with 455 *μ*L of N-methyl-2-phenylindole in acetonitrile (Reagent 1) was diluted with ferric iron in methanol. Samples were agitated; after which, 105 *μ*L 37% HCl was added, followed by incubation at 45°C for 60 min and centrifugation at 12,791 rpm for 10 min. Next, 150 *μ*L of the supernatant was added and absorbance was measured at 586 nm. The curve pattern with known concentrations of 1,1,3,3-tetramethoxypropane in Tris-HCl was used. The intra-assay CV was 8.5%.

### 2.4. 8-Isoprostane (8-IP)

The immunoassay reagent kit from Cayman Chemical Company® (Michigan, USA) was used according to the manufacturer's instructions. The limit of detection was of 0.8 pg/mL. The 8-IP assay was based on the principle of competitive binding between sample 8-IP, 8-IP acetyl cholinesterase (AChE) conjugate, and 8-IP tracer. Then, 50 *μ*L of samples or standard was added to each well and 50 *μ*L of 8-IP AChE tracer was added to all wells except the total activity and blank wells; and 50 *μ*L of 8-IP enzyme immunoassay antiserum was added to all wells except the total activity and blank wells. At once, 50 *μ*L of 8-IP antiserum was added to all wells except total activity, nonspecific binding, and blank wells. The plate was covered and incubated at 4°C for 18 h and then washed 5 times with buffer. Absorbance was read at 420 nm. The intra-assay CV was 12.5%.

### 2.5. Total Antioxidant Capacity

The evaluations of total antioxidant capacity (TAC) were made following the instructions of the kit manufacturer (Total Antioxidant Power Kit, number TA02.090130, Oxford Biomedical Research®), to obtain the concentration in mM equivalents of uric acid. The detection limit was of 0.075 mM. The samples and standards were diluted 1 : 40, and 200 *μ*L was placed in each well. The plate was read at 450 nm as a reference value, 50 *μ*L of copper solution was added, and the plate was incubated at room temperature for 3 minutes. Afterwards, 50 *μ*L of stop solution was added and the plate was read at 450 nm. The dilution factor was considered in the final result. The intra-assay CV was 7.8%.

### 2.6. CA-125

The evaluations of CA-125 were made following the instructions of the kit manufacturer (ELSA-CA 125 II Cusbio Bioassays®, France). The assay was performed on serum samples. 100 *μ*L of calibrators, control, or samples was placed in the corresponding groups of tubes. And 300 *μ*L of 125 I anti-CA-125 monoclonal antibody was added to each ELSA tube. The tubes were gently mixed with a vortex-type mixer. The tubes were incubated for 20 ± 2 h at room temperature (18–25°C). The tubes were washed, and afterwards, 3 mL of distilled water was added to each tube and then emptied again. The process was repeated twice more. Finally, the radioactivity bound to the ELSA with gamma scintillation counter was measured. The detection limit was 0.5 U/mL.

### 2.7. Statistical Analysis

Continuous variables are expressed as mean ± standard deviation (SD) or standard error of the mean (SEM) and were analyzed with nonparametric tests according to the results obtained by the Kolmogorov-Smirnov test. For the comparisons between groups, the Mann–Whitney *U* test was used, and Kruskall Wallis test for baseline–final results. The categorical variables are presented as frequencies and percentages and were analyzed with the chi^2^ test. A value of *p* ≤ 0.05 was considered statistically significant, and the confidence interval was 95%.

### 2.8. Ethical Considerations

The scientific research study abides by the regulations of the internationally established guidelines of the Declaration of Helsinki 1964, revised in October 2013 at the World Medical Assembly. All procedures were performed according to regulations stipulated in the General Health Legal Guidelines for Healthcare Research in Mexico, 2nd Title, in Ethical Aspects for Research in Human Beings, Chapter 1, Article 17, corresponding to a Category II study as research with a minimal risk, in prospective studies that involve data risks through common procedures in physical, psychological, or diagnostic examinations or routine treatments, with Registration number R-2014-1310-38. All patients gave and signed the informed consent form in the presence of signed witnesses. Patients had the right to withdraw from the study at any time without representing harm to the patient-doctor relationship and without affecting their treatment. At all times, total confidentiality was maintained, and the patients were informed of the results throughout the study.

## 3. Results

Twenty-two patients with ovarian tumor and ascites were recruited, and follow-up was 12 months. One patient was excluded due to presenting with germinal ovarian cancer, because its management requires a chemotherapy treatment scheme that differs from platinum. Then, 21 patients with OEC cancer were included. The average age of all patients included was 53.24 years, with a range of 34–73 years and a mode of 46 years.  Table 1 shows the demographic and clinical data. Baseline levels of the CA-125 antigen were measured in all groups. The platinum-refractory patients had the highest levels of the CA-125 antigen with 963.80 ± 363.80 U/mL, and because they perished prior to the end of the first year, final evaluations were not obtained. At the end of the study, the platinum-resistant patients had CA-125 antigen levels of 4211.95 ± 2105.98 U/mL despite the paclitaxel- and carboplatin-based chemotherapy. The platinum-refractory patients were found in the most advanced clinical stages (IIIC and IV), followed by the platinum-resistant (IIIB, IIIC, and IV) patients. Of the platinum-sensitive patients, 2 were in stage IIB and 4 were in stage IIIC. Malignant ascites was found in 7 platinum-sensitive, in 4 platinum-resistant, and in 7 platinum-refractory patients. Optimal cytoreduction was possible in all of the borderline patients, all of the platinum-sensitive patients, and 1 platinum-resistant patient. Suboptimal cytoreduction was possible in 3 platinum-resistant patients and 7 platinum-refractory patients. All of the platinum-sensitive and platinum-resistant patients and 1 platinum-refractory patient received the 6 complete cycles of chemotherapy with intervals of 21 days. All of the 7 platinum-refractory patients perished, 5 of them during the first chemotherapy cycle; and 2 platinum-resistant patients died during the study period (Table 1).

The analysis of the results of the markers of oxidative stress and inflammation initially included all of the patients.

### 3.1. 8-Isoprostanes

The plasma levels of 8-IP for healthy controls had 12.35 ± 1.47 pg/mL. The baseline plasma levels of the 8-IP marker were 15.13 ± 1.50 pg/mL and final 16.90 ± 1.60 pg/mL, similar to those of the healthy controls. However, in ascites fluid, the 8-IP levels were significantly increased with 117.40 ± 62.70 (*p* = 0.002) versus healthy controls and versus baseline–final results. The 8-IP plasma levels, depending on the response to platinum, were similar in all groups: platinum-sensitive had 13.60 ± 2.14 pg/mL, platinum-resistant 10.40 ± 1.70 pg/mL, and platinum-refractory had 19.20 ± 2.80 pg/mL, without a significant difference versus healthy controls. Levels of the 8-IP marker in ascites fluid were significantly elevated among the different treatment groups (*p* = 0.03): 8-IP levels in platinum-sensitive patients were 86.62 ± 26.70 pg/mL, platinum-resistant patients had 36.70 ± 23.80 pg/mL, and platinum-refractory patients had 17.10 ± 1.50 pg/mL (Table 2).

### 3.2. LPO

Plasma levels of LPO in healthy controls were 2.68 ± 0.28 *μ*M. The levels in all patients included were as follows: baseline 2.70 ± 0.30 *μ*M and final 2.60 ± 0.30 *μ*M. Findings showed elevated levels of LPO in ascites fluid with 12.60 ± 5.80 *μ*M versus healthy controls, without a significant difference (Table 3). The plasma LPO levels between the different groups of EOC patients were similar: platinum-sensitive patients had 2.70 ± 0.29 *μ*M, platinum-resistant patients had 1.78 ± 0.25 *μ*M, and platinum-refractory patients had 3.20 ± 0.78 *μ*M, without significant difference versus healthy controls (Table 4). The plasma LPO levels baseline–final did not demonstrate significant changes. The evaluation of LPO in ascites fluid among the groups treated with platinum produced significant differences (*p* = 0.05). The platinum-sensitive patients obtained 14.90 ± 9.30 *μ*M, the platinum-resistant patients, 27.10 ± 23.90 *μ*M, and the platinum-refractory patients had 3.40 ± 1.50 *μ*Mm (Table 2).

### 3.3. Total Antioxidant Capacity

The normal plasma levels of TAC in the healthy control group were 429.42 ± 61.50 mM versus the significant elevation found in the ascites fluid of all patients, 909.30 ± 78.60 mM (*p* = 0.001). In plasma, a significant decrease of TAC was found in the baseline evaluations with 294.40 ± 24.10 mM versus the amount found in ascites fluid (*p* = 0.03). The final evaluation was slightly increased with 337.80 ± 17.10 mM (Table 3). Table 4 shows the baseline plasma levels of platinum-sensitive patients with 283.80 ± 33.30 mM, platinum-resistant with 179.10 ± 18.40 mM, and platinum-refractory with 393.40 ± 31.60 mM, with a significant difference between the different groups in response to platinum (*p* = 0.015). The final results did not produce significant changes compared to baseline. A significant difference was found between plasma levels of all groups versus healthy controls (*p* = 0.007). In evaluations of TAC in ascites fluid, an increase, without significant difference, was found between the different responses to platinum-based chemotherapy (Table 2): the platinum-sensitive patients had 871.00 ± 137.90 mM, platinum-resistant had 899.90 ± 152.70 mM, and platinum-refractory had 1008.80 ± 138.90.

### 3.4. IL-6

In ascites fluid, a significant increase in the levels of IL-6 was found, with 1342.30 ± 188.90 pg/mL (*p* = 0.007), versus plasma levels of healthy controls with 448.34 ± 279.00 pg/mL. IL-6 plasma baseline levels were 703.50 ± 162.40 pg/mL (*p* = 0.03 versus ascites fluid) and final 855.90 ± 327.90. (Table 3) There were no significant differences displayed among the different groups in plasma levels of IL-6: platinum-sensitive patients had 936.40 ± 284.60 pg/mL, platinum-resistant patients had 834.20 ± 31.00 pg/mL, and platinum-refractory patients had 363.60 ± 105.00 pg/mL, without a significant difference versus healthy controls. Despite the plasma levels of IL-6 in platinum-sensitive patients being elevated at 936.40 ± 284.60 pg/mL, there were no significant differences with all the other treatment groups including the control group (Table 4). The plasma levels in baseline–final results were similar in healthy controls and among the different groups subjected to platinum-based chemotherapy. Also, IL-6 levels in ascites fluid between the different groups included in the study were increased but not different (Table 2).

### 3.5. TNF-*α*

In the general evaluation of TNF-*α*, plasma levels in healthy controls were 160.30 ± 12.70 pg/mL, with a decrease of this cytokine in ascites fluid to 120.80 ± 30.90 pg/mL. However, the overall baseline plasma levels of TNF-*α* were significantly elevated with 190.40 ± 17.90 pg/mL versus levels in ascites fluid (*p* = 0.001) (Table 3). Plasma levels of TNF-*α* were similar in healthy controls and platinum-sensitive patients with 201.10 ± 30.00 pg/mL, in platinum-resistant patients with 249.80 ± 28.50 pg/mL and the platinum-refractory patients with 145.40 ± 22.30 pg/mL (Table 4). Also, plasma levels of TNF-*α* were similar in healthy controls and in the baseline–final results of all the different types of responses to chemotherapy. In addition, a significant difference was not found in levels of this cytokine in ascites fluid in the different groups treated with platinum (Table 2).

## 4. Discussion

Ovarian cancer is the primary cause of deaths by gynecological neoplasms. According to estimations by the American Cancer Society in 2014, 21,980 new cases of EOC were expected and 14,270 deaths due to EOC [20]. In Mexico, EOC represents 4% of neoplasms, occupies the third place in cases of cancer in females after cancer of the cervix and breast, and is considered the second cause of death due to cancer [21]. The States in the Republic of Mexico with the highest incidence of EOC are Monterrey, Mexico State, and the District Capital (Mexico City) [17]. The serous subtype of EOC was the most frequently found in the present study. It should be recognized that surgery in EOC is not only the cornerstone of treatment but it also plays an important role in the histological diagnosis and staging of the tumor [22]. The majority of patients in the study presented with advanced illness when they sought medical attention; therefore, relapses of the illness were expected even with the administration of standard, adjuvant, platinum-based chemotherapy and primary cytoreductive surgery. Survival free of progression in stage III is about ~17 months, and the global average survival can reach 45 months [23]. The patients who have short intervals without treatment (platinum-resistant) or who have never been in total remission (platinum-refractory) have response rates objective to second-line chemotherapy of about ~10–15% [24].

All of the platinum-refractory patients (100%) and 2 (50%) of the platinum-resistant patients perished soon after entering the study. Serum evaluation of the CA-125 antigen is considered fundamental in the diagnosis and in changes in levels after treatment, since it is a marker of response to treatment and forms part of the management criteria to follow [25]. In the current study, the CA-125 antigen was importantly incremented in the final evaluations of the platinum-resistant patients.

One of the characteristics of EOC is the production of ascites fluid. It should be considered that ascites forms an interesting tumor microenvironment, enriched with signals that favor proliferation of the tumor through invasion and antiapoptotic molecules, and so contributes to resistance to chemotherapy and tumor heterogeneity [8]. The profile of cytokines in ascites in EOC has demonstrated the presence of protumorigenic and antitumorigenic factors in the microenvironment, with elevated levels of protumorigenic cytokines that include IL-6, IL-8, IL-10, IL-15, IP-10, MCP-1, MIP-1*β*, and the VEGF, and the significant decrease in levels of the IL-2, IL-5, IL-7, and IL-17 and the platelet-derived growth factor [26]. These factors contribute in a cumulative way to the creation of the proinflammatory and immunosuppressor microenvironment that favors tumor proliferation [27] The IL-6 and the IL-10 have received major attention owing to their correlation to poor prognosis and inadequate response to treatment [12].

In 2012, the profile of cytokines in ascites was reported in 10 patients with EOC where the greatest expressions of various inflammation regulator factors were demonstrated, including IL-6, IL-6R, IL-8, IL-10, leptin, osteoprotegerin, and the urokinase-type plasminogen activator [28]. Also, the authors demonstrated that the increase in IL-6 in ascites fluid is an independent factor of poor prognosis for EOC [29]. The role of the IL-6 contributes to the progression of EOC by inhibiting apoptosis, stimulation of angiogenesis, increasing migration, and stimulation of cellular proliferation [28].

In the present study, we found an important increase in plasma levels of IL-6 baseline–final (*p* = 0.003) in all patients included, and levels of IL-6 in ascites fluid were elevated significantly versus healthy controls, as expected (*p* = 0.007). The implication of IL-6 in the pathogenesis of EOC is well-documented: it seems the primary source of IL-6 secreted in biological fluids is produced by the tumor tissue [30]. The ovarian tumor cells produce the stimulating factor of the macrophage colonies, and this factor is a potent chemical attractor for the monocytes that stimulates the monocytes and macrophages to produce TNF-*α*, IL-1*α*, or IL-1*β*; all with the capacity to stimulate the growth of the ovarian tumor cells [31]. In the present study, we found diminished levels of TNF-*α* in ascites fluid and significant increases in plasma in the baseline evaluations in all patients.

On the other hand, ascites is also very attractive as a resource for studies in discovering other biomarkers. Here, we found a significant increase in the 8-IP marker in ascites fluid (*p* = 0.01) and in the baseline plasma evaluations (*p* = 0.02) in all of the patients included. The plasma LPOs, in all evaluations, did not reveal any significant differences, although in ascites fluid in platinum-resistant patients there was a significant increase (*p* = 0.05) of LPO versus the platinum-refractory patients who had very low levels of LPO. Interestingly, we found a significant elevation of TAC in ascites (*p* = 0.001) and a decrease in this concentration in the baseline plasma results (*p* = 0.031), which suggests an important leakage of the antioxidants in the ascites fluid. Upon searching the literature, there were no available reports on the behavior of the markers 8-IP, LPO, and TAC in plasma and ascites fluid. Ascites is a proximal fluid with the capacity to reveal events in the early stages of EOC because the concentration of soluble factors associated with cancer tends to be much higher in ascites than in serum or plasma, which makes malignant ascites a promising source for investigation of diverse diagnostic, therapeutic, and prognostic markers [32].

In conclusion, EOC is a heterogeneous neoplasm with diverse responses to standard platinum-based treatment and cytoreductive surgery, which makes it a priority to develop new prognostic markers prior to treatment that identify patients who could have poor response to standard platinum-based chemotherapy.

The limitations of the study are based on the small number of patients included and the short length of follow-up.

## Figures and Tables

**Table 1:** Ovarian cancer clinical data. A predominance of ovarian serous cystadenocarcinoma with malignant ascites can be observed. Cytoreduction was optimal in 14 patients and suboptimal in 10 patients: only 10 patients were platinum-sensitive, 4 platinum-resistant, and 7 platinum-refractory (all 7 perished during the first year). The majority of patients were discovered in advanced stages.

	Platinum-sensitive	Platinum-resistant	Platinum-refractory
Weight (kg)	69 ± 19	75 ± 25	46 ± 21
Body mass index (BMI)	27 ± 7	30 ± 9	21 ± 4
Ag CA-125 baseline U/mL	607.37 ± 183.13	915.8 ± 373.87	963 ± 363.80
Ag CA-125 final U/mL	21.87 ± 6.59	4211.95 ± 2105.98	62.6 ± 25.56
Clinical stage			
IC	2		
IIB	2		
IIIB	2	1	
IIIC	4	2	5
IV		1	2
Histology	9 Cystadenocarcinoma	3 Cystadenocarcinoma	6 Cystadenocarcinoma
	1 Undifferentiated	1 Endometrioid type	1 Endometrioid type
Ascites	3 Positive		
Malignant ascites	7 Positive	4 Positive	7 Positive
Cytoreduction	10 Optimal	3 Suboptimal 1 Optimal	7 Suboptimal
Cycle frequency days	21	21	5–1 cycle
			1–6 cycles
			1-2 cycles
Carboplatin (mg)	570 ± 109	471 ± 187	464 ± 124
Paclitaxel (mg)	300 ± 39	273 ± 106	254 ± 63
Deceased		2	7

**Table 2:** Oxidative and inflammatory status in ascites due to ovarian cancer. The significant difference between study groups treated with platinum and the concentrations of LPO and 8-IP in ascites fluid is noteworthy.

	Platinum-sensitive	Platinum-resistant	Platinum-refractory	*p* ^∗^ (K-W)
*Antioxidant*				
TAC mM trolox	871.00 ± 137.90	899.90 ± 152.70	1008.80 ± 138.90	0.60
*Oxidants*				
LPO *μ*M	14.90 ± 9.30	27.10 ± 23.90	3.40 ± 1.50	**0.05** ^∗^
8-IP pg/mL	86.62 ± 26.70	36.70 ± 23.80	17.10 ± 1.50	**0.03** ^∗^
*Proinflammatory cytokines*				
IL-6 pg/mL	1582.60 ± 346.10	969.60 ± 76.30	1382.30 ± 257.60	0.31
TNF-*α* pg/mL	146.10 ± 62.80	102.00 ± 27.50	75.10 ± 17.90	0.52

TAC: total antioxidant capacity; LPO: lipoperoxides; 8-IP: isoprostanes; IL-6: interleukin-6; TNF-*α*: tumor necrosis factor alpha; K-W: Kruskall-Walis test. ^∗^Comparison between treatment response groups.

**Table 3:** Oxidative and inflammatory state in ovarian cancer. A significant increase of the 8-IP marker in ascites fluid versus baseline plasma levels can be observed. Also, an important leakage of antioxidants (TAC) in ascites fluid compared to plasma levels of healthy controls and the baseline TAC evaluations. A significant increase of IL-6 in ascites fluid versus baseline plasma levels was found. The TNF-*α* was significantly diminished in ascites and elevated in baseline evaluations.

	Healthy control plasma	Ascites	^Ŧ^ *p* = HC versus ascites	^¥^ *p* = HC versus baseline	Plasma		^¤^ *p* = baseline–final	^§^ *p* = plasma baseline versus ascites
					Basal	Final		
*Oxidants*								
8-IP pg/mL	12.35 ± 1.47	117.40 ± 62.70	**0.01**	0.48	15.13 ± 1.50	16.90 ± 1.60	0.14	**0.002**
LPO *μ*M	2.68 ± 0.28	12.60 ± 5.80	0.50	0.56	2.70 ± 0.30	2.60 ± 0.30	0.26	0.11
*Antioxidant*								
TAC mM trolox	429.42 ± 61.50	909.30 ± 78.60	**0.001**	**0.03**	294.40 ± 24.10	337.80 ± 17.10	0.19	**<0.001**
*Proinflammatory cytokines*								
IL-6 pg/mL	448.34 ± 28.00	1342.30 ± 188.90	**0.007**	0.42	703.50 ± 162.40	855.90 ± 327.90	0.31	**0.003**
TNF-*α* pg/mL	160.30 ± 12.70	120.80 ± 30.90	0.06	0.36	190.40 ± 17.90	164.40 ± 34.22	0.18	**0.001**

TAC: total antioxidant capacity. ^Ŧ^Healthy control (HC) versus ascites Mann–Whitney *U* test. ^¥^HC versus baseline plasma Mann–Whitney *U* test. ^**¤**^Baseline–final Wilcoxon test. ^§^Baseline plasma versus ascites Mann–Whitney *U* test.

**Table 4:** Oxidative and inflammatory status in plasma due to ovarian cancer in all patients. Noteworthy are the levels in healthy controls versus the study groups and the significant difference depending on the response to platinum in relation to total antioxidant capacity.

	Healthy control	Platinum-sensitive	Platinum-resistant	Platinum-refractory	*p* ^∗^ (K-W)	*p* ^∗∗^ (K-W)
*Antioxidant*						
TAP mM trolox	429.42 ± 61.50	283.80 ± 33.30	179.10 ± 18.40	393.40 ± 31.60	**0.007**	**0.015**
*Oxidants*						
8-IP pg/mL	12.35 ± 1.47	13.60 ± 2.14	10.40 ± 1.70	19.20 ± 2.80	0.26	0.22
LPO *μ*M	2.68 ± 0.28	2.70 ± 0.29	1.78 ± 0.25	3.20 ± 0.78	0.38	0.32
*Proinflammatory cytokines*						
IL-6 pg/mL	448.34 ± 28.00	936.40 ± 284.60	834.20 ± 31.00	369.60 ± 105.00	0.44	0.33
TNF-*α* pg/mL	160.30 ± 12.70	201.10 ± 30.00	249.80 ± 28.50	145.40 ± 22.30	0.27	0.21

^∗^Comparison between the study groups with the healthy control. ^∗∗^Comparison between the study groups. TAC: total antioxidant capacity; LPO: lipoperoxides; 8-IP: isoprostanes; IL-6: interleukin-6; TNF-*α*: tumor necrosis factor alpha; K-W: Kruskall Wallis test.
